# Fresh Hemorrhages in Intestinal Resection Margins Are Associated with Unfavorable Clinical Outcomes in Preterm Infants with Necrotizing Enterocolitis with Surgical Intervention

**DOI:** 10.3390/life14111510

**Published:** 2024-11-20

**Authors:** Ole Schickedanz, Florian Friedmacher, Steffen Gretser, Yannick Braun, Peter Johanes Wild, Udo Rolle, Elise Gradhand

**Affiliations:** 1 Dr. Senckenberg Institute of Pathology, University Hospital Frankfurt, Goethe University Frankfurt, Theodor-Stern-Kai 7, 60590 Frankfurt, Germany; ole.schickedanz@t-online.de (O.S.); steffen.gretser@unimedizin-ffm.de (S.G.); peter.wild@ukffm.de (P.J.W.); 2 Department of Pediatric Surgery, University Hospital Frankfurt, Goethe University Frankfurt, 60590 Frankfurt, Germany; florian.friedmacher@unimedizin-ffm.de (F.F.); y.braun@kinderkrebsstiftung-frankfurt.de (Y.B.); udo.rolle@unimedizin-ffm.de (U.R.)

**Keywords:** necrotizing enterocolitis, fresh hemorrhages, pediatric pathology, pediatric surgery

## Abstract

Background: Necrotizing enterocolitis (NEC) is a common disease in premature infants. If conservative treatment does not respond, surgical removal of the necrotic section of bowel is common practice. This study investigates whether there is a correlation between the histopathological findings and the postoperative clinical course of the children who have undergone surgery. To date, only a few detailed studies on a possible correlation have been published. Methods: The surgical specimens of 49 infants diagnosed with NEC in the years 2010–2019 were re-evaluated. The histologic specimens were examined for tissue viability and hemorrhage of the resection margins, peritonitis or perforation of the central resection segment. The groups were divided according to the clinical outcome: death, postoperative complications and patients without complications. Results: 5 of 49 (10.2%) children died, 22 children (44.9%) required reoperation, while 22 (44.9%) had no complications. Univariate and multivariate analyses showed a significant association between fresh hemorrhage in the resection margins and postoperative outcome. In our data, correlation between the vitality of the resection margins or the extent of necrosis and the postoperative course was not seen. Conclusion: This retrospective study shows a significant correlation between a fresh hematoma in the tissue of the resection margin and the clinical prognosis. Fresh bleeding in the resection margins was associated with increased morbidity with stenosis and possibly another surgical intervention. In contrast, no further correlation was found between the vitality of the tunica mucosae, the tunica muscularis or peritonitis in the resection margin or central part of the NEC specimen and the clinical course. In summary, it can be said that the presence of a fresh hematoma at the resection margin is significantly linked to a poorer clinical outcome for the infants with NEC surgery. Other histopathological findings of the surgical specimen with regard to the clinical course show now significant correlation and, therefore, the histological examination mainly serves the medico-legal documentation and quality assurance of the NEC operation.

## 1. Introduction

**Figure 1:**
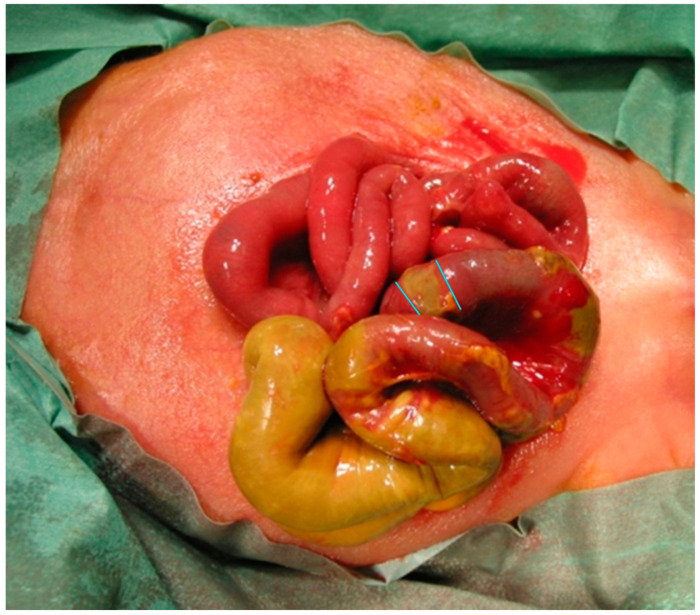
Intraoperative findings of multiple, segmental NEC. The blue lines mark potential resection margins.

Necrotizing enterocolitis (NEC) is one of the most common and devastating gastrointestinal emergencies in neonates, predominantly affecting premature and low-birth-weight infants in the first weeks of life [1,2,3,4]. There seems to be a marked variation in global NEC incidence rates, currently ranging between 2% and 22% in high-income countries [5,6,7]. The pathogenesis of NEC remains poorly understood but is believed to have multifactorial causes, related to intestinal immaturity, mesenteric hypoxia/ischemia, microbial dysbiosis and several other predisposing factors [8,9,10,11,12]. The resulting inflammation and eventual necrosis of the NEC-affected intestine frequently leads to sepsis, multiorgan failure and death [13,14]. While approximately half of all neonates with acute NEC respond to gastric decompression, bowel rest, broad-spectrum antibiotics and supportive medical measures, many develop progressive disease eventually requiring surgical intervention [15,16] (see Figure 1. Despite significant efforts at prevention and treatment [17], NEC-related mortality continues to be high, ranging between 12% and 32%, which can reach 50% in surgical cases [18,19,20,21,22,23]. Moreover, poor clinical outcomes and substantial long-term morbidity were reported in up to 50% of patients with NEC, particularly in more severe cases and those requiring surgery with resection of necrotic bowel [24,25,26,27]. The histopathological features of the resected intestine in NEC have been well-described, including frank gangrene, coagulative necrosis, bacterial overgrowth/invasion with signs of pneumatosis intestinalis or perforation, often resembling ischemic changes, which occur presumably due to alterations in microvascular function [28,29,30]. Although the resected bowel specimens are typically sent for pathological evaluation following laparotomy for acute NEC, prognostic information obtained from the histological examination is largely uncertain. This is mostly due to the fact that the severity and extent of these necrotic lesions appear to be highly variable with a paucity of qualitative studies that specifically examined clinicopathological relationships in NEC [31,32,33]. Therefore, the objective of this study was to investigate whether there is an association between the histopathological features and the occurrence of postoperative complications in preterm infants with acute NEC who underwent surgical bowel resection, thus providing important information on the potential further clinical outcomes.

## 2. Material and Methods

### 2.1. Patient Population and Study Design

With appropriate institutional ethical committee approval (ethic committee of the university hospital Frankfurt, Nr.: 2021-123), a retrospective cohort study was performed. It consists of all premature neonates with definite (i.e., Bell’s stage II or III) and histologically confirmed NEC who underwent exploratory laparotomy with bowel resection and/or further surgical intervention for NEC-related complications at a large, tertiary-level referral hospital from 1 January 2010 to 31 December 2019. The respective decisions for surgery were each based on a multidisciplinary team approach considering clinical, laboratory and radiological findings. In cases of multiple NEC lesions in close proximity, the affected bowel was resected as a whole specimen, whereas in skip lesions that were separated by longer viable bowel segments, only the affected bowel was resected and anastomosed. Patients with coexisting anomalies (e.g., congenital heart defect) or any histopathological diagnosis other than NEC but a similar clinical presentation (e.g., focal intestinal perforation, meconium ileus or volvulus) were excluded from this study, which was conducted according to the principles expressed in the 1964 Declaration of Helsinki and its later amendments. Each case was identified by using the hospital inpatient enquiry system and relevant data were extracted from the individual medical and operative records. General patient characteristics including sex, gestational age, birth weight, singleton/multiple pregnancy and age at surgery were noted (Table 1). In addition, microbiological results from intraoperative abdominal swabs and blood cultures were collected. The clinical outcome was the occurrence of postoperative complications requiring surgical revision within one year, such as NEC recurrence, clinical deterioration with prolonged ileus despite maximal medical therapy and development of intestinal strictures and death (Table 2).

### 2.2. Histopathological Evaluation

**Figure 2:**
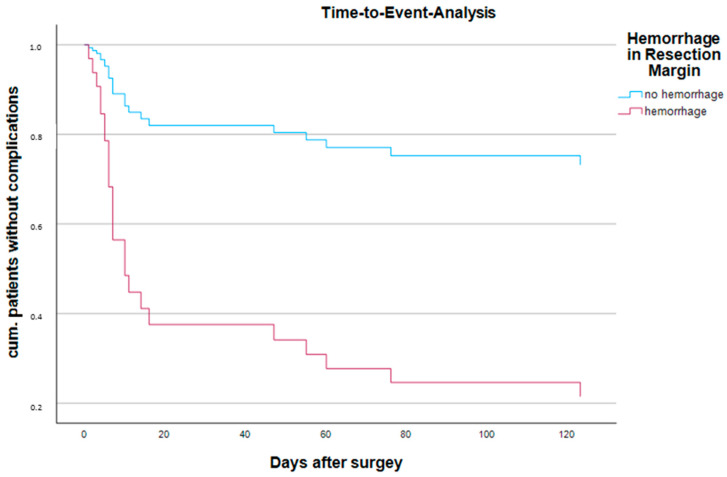
Time-to-event analysis. Hemorrhagic vs. no hemorrhagic in resection margin.

Two independent, board-certified pathologists unaware of any clinical information of included NEC patients evaluated all the available surgical specimens. The formalin-fixed, paraffin-embedded intestinal tissue was processed and cut in a standardized fashion, followed by hematoxylin and eosin staining. In each case, serial sections from both the proximal and distal resection edges as well as from the central area of NEC-affected bowel were investigated under a ZEISS Axio Scope.A1 light microscope (Carl Zeiss Microscopy GmbH, Göttingen, Germany). At least ten high-power fields (40× objective) per section were analyzed for histopathological evidence of NEC, including inflammation, necrosis and perforation, as well as presence of hemorrhages or strictures. Figures 2, 4 and 5 show representative histopathological findings in surgically resected NEC intestine. The severity of NEC was assessed by the depth at which these histopathological features occurred. Inflammation was defined as the infiltration of monocytes/granulocytes and/or macrophages/neutrophils, thus CD15 (monocyte/granulocyte marker, Dako Anti-CD15 Monoclonal Mouse FLEX Clone Carb 3) and CD68 (macrophage, Dako Anti-CD68 Monoclonal Mouse FLEX Clone KP1) staining was used to quantify the extent of acute or chronic inflammatory reaction. Intestinal tissue with a regular four-layer structure, visible hematoxylin staining of the nuclei and well-differentiated cell borders was considered as vital, whereas tissue with fading cell clusters, absent or faded nuclei, diminished cytoplasmic eosin staining and blurred cell borders was regarded as avital. In the presence of localized patchy necrosis, the mucosal, submucosal or muscular layer appeared poorly demarcated or partially absent. If necrosis was present in all the tissue layers, either focally and/or extensively, this was classified as transmural necrosis. A perforation was characterized by full-thickness necrosis with interruption of the intestinal wall continuity, in which the perforation margins appeared blurred and rounded. If fibrin and neutrophils were seen on the serosal surface, peritonitis was diagnosed. Iron staining was applied to estimate the age of hemorrhages. Extravasation of erythrocytes into the submucosal and/or subserosal bowel layers were viewed as fresh hemorrhages. Extensive hemosiderin deposition and hemosiderin-laden macrophages in the submucosa and/or subserosa were a sign of previous hemorrhage that had occurred several days, weeks or months ago (i.e., the more pronounced the infiltration of chronic inflammatory cells within the first week after tissue trauma, the older the hemorrhage) [34]. A significant narrowing of the intestinal lumen due to submucosal fibrosis was considered as a NEC stricture. Any discrepancies between the pathologists were resolved by mutual consensus.

### 2.3. Statistical Analysis

All data were extracted into an Excel spreadsheet and statistical analysis was carried out using IBM SPSS Statistics 28.0 software package (IBM Corp., Armonk, NY, USA). Results are presented as median with interquartile range (IQR) or number (*n*) with percentage (%), as appropriate. Fisher–Freeman–Halton test was used to determine whether there was a statistically significant association between two categorical variables (i.e., histopathological features and primary or secondary clinical outcome measures). A binary logistic regression analysis was then performed to identify histopathological risk factors predicting the occurrence of postoperative complications with need for further surgical intervention or death. Goodness-of-fit of the logistic regression model was assessed using Hosmer–Lemeshow test. Additionally, a time-to-event analysis was conducted in order to ascertain the median time with 95% confidence interval (CI) between the initial operation and the occurrence of postoperative complications requiring further surgery or death depending on the presence of relevant histopathological features. Overall, the significance level was assumed to be 0.05.

## 3. Results

### 3.1. General Patient Characteristics and Postoperative Outcomes

Overall, 49 preterm infants with histologically confirmed NEC (i.e., Bell’s stage II or III) were included in this study. Thirty-one (63.3%) were male and 18 (36.7%) were female. The median gestational age was 27 (IQR, 25–29) weeks with a median birth weight of 810 (IQR, 680–1050) g. Of these, 40 (81.6%) derived from singleton pregnancies, whereas the remaining nine were from twin (*n* = 8) or triplet (*n* = 1) pregnancies, respectively. The median age at the time of first surgery for NEC with bowel resection was 16 (IQR, 8–31) days. Postoperative follow-up was one year. During this time, 22 (44.9%) patients had an uneventful recovery, while 22 (44.9%) developed NEC-related complications with the need for reoperation due to ongoing clinical deterioration (*n* = 18), development of intestinal strictures (*n* = 3) and NEC recurrence (*n* = 1). In total, five (10.2%) patients died within 1 year after the first surgical intervention (see Table 2). Microbiological results from intraoperative abdominal swabs were positive in 29 (59.2%) cases and blood cultures in three (6.1%).

### 3.2. Histopathological Findings

A total of 135 intestinal specimens resected from the included 49 infants with NEC were histologically evaluated with serial sections from the proximal and distal resection edges as well as from the most affected central area (Table 3). Tissue samples from surgical revisions due to NEC-related complications were available from 16 (72.7%) of the 22 patients who had been re-operated. Six patients (6/22, 273%) had no tissue removed and, therefore, were not submitted for histology. While the mucosal and muscular layers in both resection edges appeared vital in 30 (61.2%) and 40 (81.6%) of the NEC specimens; this was not the case in 19 (38.8%) and 9 (18.4%), respectively. Transmural necrosis, peritonitis and hemorrhages were identified in 9 (18.4%), 15 (30.6%) and 22 (44.9%) of the resection margins. Subsequent CD15 and CD68 staining of the 22 proximal and distal resection margins with hemorrhages detected a mild, moderate or distinct infiltration of monocytes/granulocytes and macrophages/neutrophils in all samples. Iron staining did not show any hemosiderin-loaded macrophages in resection edges with hemorrhages in 16 (72.7%) of the 22 specimens, whereas staining was discreet in five (22.7%) and mild in one (4.6%). Thus, there was a co-localization of monocytes/granulocytes with macrophages/neutrophils in all cases, while a co-localization of monocytes/granulocytes, macrophages/neutrophils and hemosiderin-loaded macrophages was noticed in only five (22.7%) instances. Conversely, the mucosal and muscular layers in the most affected central area were considered to be non-vital in 21 (42.9%) and 33 (67.3%) of the NEC samples with transmural necrosis in 21 (42.9%), perforation in 23 (46.9%) and peritonitis in 22 (44.9%) cases. A microscopically evident narrowing of the intestinal lumen due to submucosal fibrosis was found in 11 (22.4%) specimens from the central area of previously NEC-affected bowel with re-surgery (see also Table 3).

### 3.3. Association Between Histopathological Features and Occurrence of Postoperative Complications or Death

**Figure 3:**
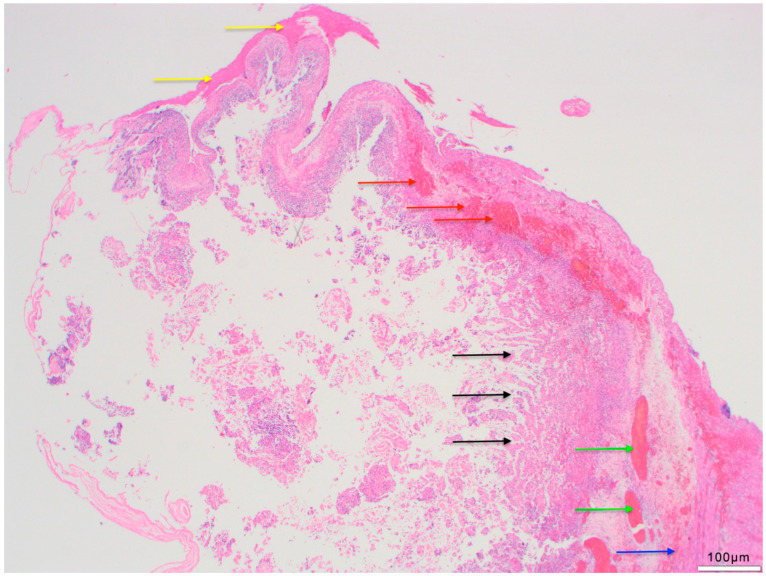
Histopathological findings of NEC. Avital mucosa (black arrow). Partly preserved muscularis (blue arrow). Congested blood vessels (green arrow). Left side of the picture: full-wall necrosis without recognisable stratification partly perforated. Peritonitis at the top of the picture (yellow arrow). Hemorrhage within the submucosa and muscularis propria (red arrows). (H&E; 2.5×).

**Figure 4:**
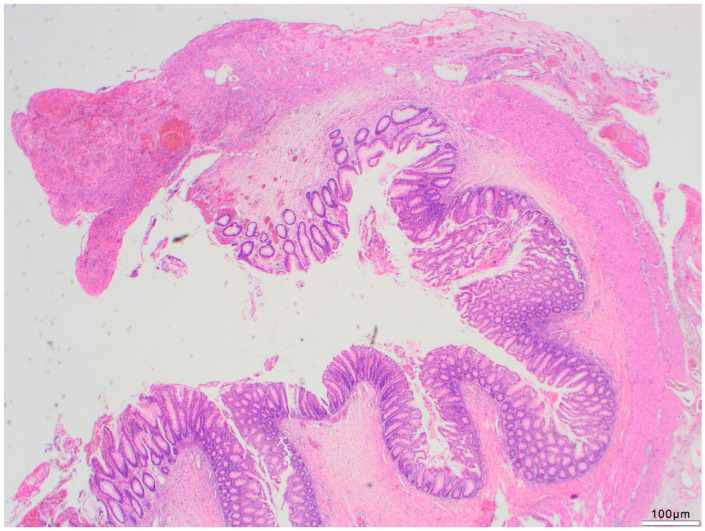
Antimesenterial full thickness ischemia (black arrow) and perforation (green arrow). (H&E; 2.5×).

**Figure 5:**
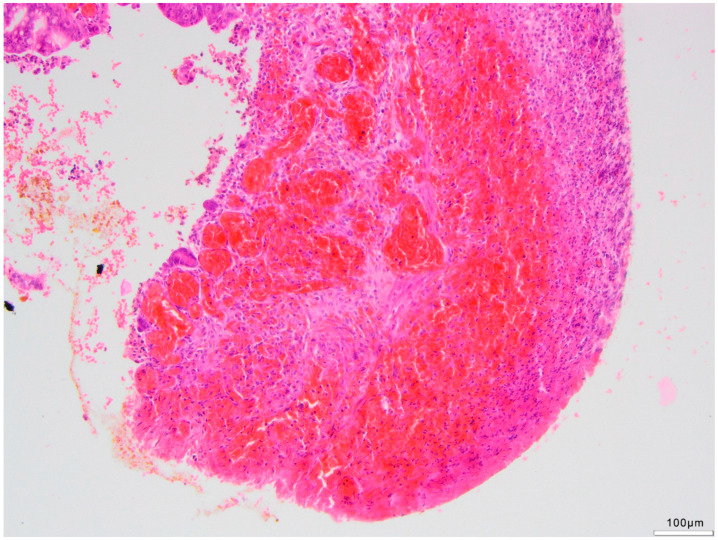
Fresh hemorrhage at resection margin with congested vessels (black arrow) and diffuse hemorrhage within the lamina propria and muscularis propria (green arrow). (H&E, 4×).

There were no obvious histopathological differences in NEC severity between survivors and those who died. Statistical analysis demonstrated that there was no significant association between tissue viability, the presence of transmural necrosis or peritonitis in resection edges, and the occurrence of postoperative complications with the need for further surgical intervention or death. While fresh hemorrhages (Figure 2) in the resection edges were statistically significantly associated with the occurrence of postoperative complications requiring surgical revision and death, no statistically significant association was found for CD15, CD68 and iron staining (Table 4 and Table 5). Similarly, none of the histopathological features in the NEC-affected central areas (i.e., tissue viability, transmural necrosis, perforation, peritonitis and strictures) were statistically significantly associated with the occurrence of postoperative complications with need for surgical intervention or death (Table 3). The binary logistic regression model confirmed that only fresh hemorrhages in the proximal and distal resection edges of resected NEC specimens had a statistically significant effect in predicting the occurrence of postoperative complications that require further surgical intervention, while this was not the case for the other histopathological features (Table 3, Figure 3, Figure 4 and Figure 5). Table 2 illustrates the varying frequency of postoperative complications with the need for additional surgery within one year depending on the presence of fresh hemorrhages in both resection edges. In NEC patients with fresh hemorrhages in their proximal and distal resection edges, postoperative complications or death occurred after a median of 10 [95% CI (6.5–13.5)] days. No time could be estimated for NEC patients without hemorrhages in their resection edges as postoperative complications or death occurred in less than 50% of cases. The results of the log-rank test revealed a significant difference between NEC patients with and without hemorrhages in their proximal and distal resection edges [χ^2^(1) = 13.08; *p* < 0.001].

### 3.4. Binary Logistic Regression Model

In the group of NEC patients, the binary logistic regression model with the postoperative course as the dependent variable and the histopathological findings of the resection margins as the independent variable was statistically significant χ^2^(5) = 15. 957, *p* = 0.007 with an acceptable variance explanation of Nagelkerke’s R^2^ = 0.371. The goodness-of-fit test in the Hosmer–Lemeshow test showed a high goodness of fit, χ^2^(8) = 7.501 *p* > 0.05.

Of the five variables included in the model, one was significant, hemorrhage (*p* = 0.002) (Figure 2), while the vitality of the mucosa (*p* = 0.192), the vitality of the muscularis (*p* = 0.714), full wall necrosis (*p* = 0.168) and peritonitis (*p* = 0.502) had no significant effect on the predictive performance of the model. Hemorrhage was more likely to lead to complications with an Exp(B) of 12.806 (95% CI = 2.54–64.62).

## 4. Discussion

This study demonstrates that there is a statistically significant association between the clinical course of preterm infants and perioperative fresh hemorrhage into the tissue of the resection margin of an NEC-resection specimen.

To date, very few studies exist, such as those by Eaton et al. [32] and Remon et al. [31], that have investigated a relationship between the histopathologic findings of NEC and the postoperative clinical course.

According to Remon et al., the depth of bacterial invasion into the intestinal wall can be used to predict the mortality of operated children with NEC. Obvious bacterial colonization was not observed in any of our studied specimens. According to Eaton et al., a weak correlation exists between the extent of histologically detected intestinal necrosis and patient survival. The approach of the studies differs from this study in many ways.

For example, Eaton et al. attempted to determine the relationship between severe damage to intestinal tissue (full-thickness necrosis, mucosal necrosis and no necrosis) and survival and non-survival. Here, as in our study, there was no correlation between the extent of necrosis and the vitality of the resected tissue with clinical outcome [32]. Ultimately, the extent of necrosis within the resected segments appears to be irrelevant, as the goal of surgical intervention is to cure the NEC by removing the necrotic and non-vital bowel segments. This work shows that the clinical macroscopic impression seems to be sufficient to identify the transition from the vital to the non-vital bowel segment.

In this study, the outcome of the children was analyzed not only for survival or demise but also included complications as a possible endpoint. This more detailed approach allowed us to identify hemorrhage as an independent risk factor for re-surgery. With these data, it was shown that there is a strong correlation between the presence of fresh hemorrhage into the tissue of the resection margin and the postoperative clinical course. A pathophysiological explanation for the discovered association could be that the hemorrhage and the extent of the hematoma contribute to an enhanced immune response due to tissue damage by the hematoma through displacement and the clearance response to tissue damage [35] with scarring and consequent shrinkage and thus stenosis. It is conceivable that normally physiologic formation of granulation tissue [36] in the preterm infant could lead to excessive, increased fibrosis of the remaining intestine at the surgical site by a dysregulated immune response [37] to degradation products of erythrocytes and other blood components, resulting in defect healing with scarring.

A possible conclusion for pediatric surgeons could be to operate as atraumatically as possible and, in the case of macroscopically visible hematomas and hemorrhages, to remove them at the same time at the initial surgery in order to obtain a hematoma-free resection margin with maybe even a supportive intraoperative frozen section.

In the further course of the study, it was analyzed whether the extent of an inflammatory reaction because of hemorrhage in the resection margin can be verified and quantified and whether a possible conclusion can be drawn from this due to a possible correlation with the postoperative course. In the specimens from the initial surgery, only fresh hematomas were seen with absent to minimal evidence of iron and only focal resident macrophages, suggesting that the hematoma occurred intraoperatively as a result of intraoperative tissue manipulation and did not exist before surgery. In only five specimens, iron staining revealed single hemosiderin-laden macrophages in the submucosa as an indication of possible minor prior hemorrhage or previous ischemic injury.

All specimens from patients with hemorrhage in the resection margin showed low numbers of CD15-labeled monocytes and granulocytes and CD68-expressing macrophages. This corresponds most closely to the physiological resident cell population of the submucosa [38]. The correlation analysis between CD15 and CD68 as well as iron staining and the postoperative course showed no statistical correlation (*p*-value always > 0.05). A specific correlation analysis between the findings of the CD15, CD68, and iron stains of the initial surgery with the presence of stenosis in the clinical course was not possible because too few specimens of the second surgeries were available. Thus, there is no recommendation for regular staining of the incision specimens by CD15, CD68, or iron.

In this work, all cases with hemorrhage within the resection margin at initial surgery showed co-localization of histiocytes and monocytes or granulocytes without hemosiderin-laden macrophages.

According to the literature, hemosiderin-laden macrophages are not detected in soft tissue before 3 days after hemorrhage. It is also known that in a cutaneous hematoma, the extent of macrophage infiltration increases with time within the whole hematoma [34].

Only five cases (23.8%) showed co-localization of histiocytes, monocytes, and hemosiderin-laden macrophages. In the five cases in which hemosiderin-loaded macrophages were found, the hemorrhages can be interpreted as preoperative bleeding residuals, whereas in the 16 cases without hemosiderin-laden macrophages, fresh bleeding during NEC surgery can be assumed.

The pattern of tissue damage observed within the NEC specimen corresponds to an ischemic pattern with a punctum maximum in the antimesenteric portion of the intestinal wall (Figure 3) and with onset of microcirculatory damage in the luminal portion of the mucosa and successive damage up to the outer muscularis propria to complete wall necrosis (Figure 3 and Figure 4). These findings were also observed by Zhang et al. [39]. However, in another study, Remon et al. attempted to quantify the extent and depth of necrosis, inflammation, and bacterial invasion within resected intestinal tissue [31].

They demonstrated that there is a correlation between the presence of transmural necrosis with bacterial overgrowth and the outcome of the children. Nevertheless, the histopathological findings of this study did not show bacterial colonization. To look for the presence of bacteria using five representative cases with full-thickness necrosis. For identification, we stained the sections by Gram stain and indeed demonstrated focal bacterial colonization in one NEC case. However, this more closely resembled postmortem colonization without cellular tissue reaction, as is often the case after the autolysis of tissue. In the remaining vital sections of this single case with evidence of bacterial colonies and the remaining four representative cases, no bacterial colonization could be detected. Furthermore, the detection of bacteria by light microscopic examination of HE sections is of limited value and, according to Ring et al., only has a sensitivity of 0.43 if the bacterial aggregates are >10 µm [40]. Accordingly, another correlation found by Remon et al. between the depth of bacterial invasion and clinical outcome [31] should not be considered a clear clinical cause, but rather a sign of the extent of tissue necrosis.

The absence of bacterial growth within the resection specimens is also a result of the initial conservative treatment with antibiotics and, therefore, at the time of surgery, the bacterial colonies cannot be identified. Therefore, the initial most likely bacterial damage of the immature bowel is not seen at the stage of surgery. At the time of surgery, the leading injury or injury pattern that is left is of a mostly ischemic nature.

Apart from the only very focal presence of bacteria in full-thickness necrosis, these data did not demonstrate a correlation between the presence of transmural necrosis in the resection margin or the extent of bowel damage in the central portion of the resection specimen or even acute peritonitis with an increased mortality or even morbidity. Therefore, surgical resection alone was curative.

## 5. Conclusions

The clinical intraoperative impression and the purely macroscopic selected resection margin by the pediatric surgeon seem to be sufficient to significantly improve the outcome of the patients with NEC. This study could show that identifying fresh hematomas at the resection margin of the bowel specimen, which occurred intraoperatively, should be resected to achieve a hematoma-free resection margin for anastomosis. A recommendation for the frozen section examination to exclude a major fresh hematoma in the resection margin could be offered. However, it should be considered that this might unnecessarily prolong anesthesia and the surgical procedure. An interdisciplinary benefit assessment should be performed here. Due to the statistical correlation between the presence of hemorrhages in the resection margin and the postoperative course, the pediatric surgeons are recommended to operate as atraumatically as possible and, in case of macroscopically well visible hematomas and hemorrhages, to remove them during the initial surgery. The histological vitality of the resection margin alone is not decisive for the survival or co-morbidity of the child.

## Figures and Tables

**Table 1:** Clinical characteristics of included patients.

Patients	59
Gestational age (weeks)—median (iqr)	27 (25–29)
Birth weight (g)—median (iqr)	810 (680–1050)
Age at surgery (days)—median (iqr)	16 (8–31)
Sex (male/female)	37/22 (62.7%/37.3%)
Multiple pregnancy—*n* (%)	
Singleton	48 (81.4%)
Twins	10 (16.9%)
Triplets	1 (1.7%)

**Table 2:** NEC patients and postsurgical courses.

DIAGNOSIS	Number of Patients N (%)
**Necrotizing enterocolitis**	**49** (83.1%)
- No postsurgical complications	- 22
- **POSTSURGICAL COMPLICATIONS**	- 22
○ **FAILURE OF CLINICAL IMPROVEMENT DESPPITE MAXIMAL THERAPY**	○ 13
○ **ILEUS**	○ 8
○ **NEC RECURRENCE**	○ 1
- Deaths	- 5

**Table 3:** Histopathological findings of all NEC cases and correlation analysis with postsurgical courses.

**Histopathological Findings of the Surgery Margin of All NEC Patients (*n* = 49)**				
	No complications	Complications	Deaths	*p*-value
Vital Mucosa	15	12	3	1.000
Avital Mucosa	9	8	2	
Vital Muscularis	21	16	3	0.304
Avital Muscularis	3	4	2	
No transmural necrosis	21	14	5	0.217
Transmural necrosis	3	6	0	
No haemorrhage	20	6	1	<0.001
haemorrhage	4	14	4	
No Peritonitis	17	15	2	0.406
Peritonitis	7	5	3	
**Histopathological Findings of the Central Portions of All NEC-Patients (*n* = 49)**				
	No complications	Complications	Deaths	*p*-value
Vital Mucosa	15	10	3	0.772
Avital Mucosa	9	10	2	
Vital Muscularis	10	4	2	0.283
Avital Muscularis	14	16	3	
No transmural necrosis	13	11	4	0.654
Transmural necrosis	11	9	1	
No Perforation	11	13	2	0.388
Perforation	13	7	3	
No Peritonitis	13	11	3	1.000
Peritonitis	11	9	2	
No Stenosis	21	14	3	0.214
Stenosis	3	6	2	
**Total**	24	20	5	49

**Table 4:** Binominal logistic regression. Note: degrees of freedom were 1 for all Wald statistics.

	B	Exp(B)	Wald	*p*	95% CI for Exp(B)	
					Lower	Upper
Vitality mucosa	−1.258	0.284	1.700	0.192	0.043	1.884
Vitality muscularis	0.426	1.531	0.134	0.714	0.156	15.004
Full thickness necrosis	1.254	3.506	1.898	0.168	0.589	20.884
Peritonitis	−0.531	0.588	0.451	0.502	0.125	2.770
Hemorrhaege	2.550	12.806	9.533	0.002	2.538	64.624
Constant	−0.962	0.382	2.982	0.084		

**Table 5:** Correlation analysis CD15-, CD68- and iron staining of the specimen with hemorrhage in the resection margin.

Correlation Analysis CD15, CD68 and Iron Staining with Postoperative Course					
CD15	No Complications	Complication	Death		*p*-Value
Discreet	2	7	1	10 (17%)	0.403
Mild	1	4	1	6 (38%)	
Distinct	3	1	1	6 (38%)	
**CD68**					
Discreet	2	7	0	9 (43%)	0.296
Mild	2	4	2	8 (38%)	
Distinct	2	1	1	4 (19%)	
**Iron**					
Not detectable	5	9	2	16 (76%)	0.429
Discreet	1	3	0	4 (19%)	
Mild	0	0	1	1 (5%)	
**Total**	6	12	3	21	

## Data Availability

The data set will be available on reasonable request.
